# Regulation of *MYO18B* mRNA by a network of C19MC miRNA-520G, IFN-γ, CEBPB, p53 and bFGF in hepatocellular carcinoma

**DOI:** 10.1038/s41598-020-69179-5

**Published:** 2020-07-23

**Authors:** Goodwin G. Jinesh, Marco Napoli, Hayley D. Ackerman, Payal M. Raulji, Nicole Montey, Elsa R. Flores, Andrew S. Brohl

**Affiliations:** 1 Department of Molecular Oncology, H. Lee Moffitt Cancer Center & Research Institute, Tampa, FL 33612 USA; 2 Sarcoma Department, H. Lee Moffitt Cancer Center & Research Institute, Tampa, FL 33612 USA; 3 Cancer Biology and Evolution Program, H. Lee Moffitt Cancer Center & Research Institute, Tampa, FL 33612 USA; 4 Chemical Biology and Molecular Medicine Program. 12902 USF Magnolia Drive, H. Lee Moffitt Cancer Center & Research Institute, Tampa, FL 33612 USA

**Keywords:** Cancer, Cell biology, Computational biology and bioinformatics, Genetics, Immunology, Molecular biology, Stem cells, Diseases, Gastroenterology, Medical research, Oncology, Risk factors

## Abstract

*MYO18B* has been proposed to contribute to the progression of hepatocellular carcinoma (HCC). However, the signals that govern *MYO18B* transcription are not known. Here we show that, a network of C19MC miRNA-520G, IFN-γ, CEBPB and p53 transcriptional-defects promote *MYO18B* mRNA expression in HCCs. IFN-γ by itself suppresses *MYO18B* transcription, but promotes it when miRNA-520G is stably overexpressed. Similarly, CEBPB-liver-enriched activator protein (LAP) isoform overexpression suppresses *MYO18B* transcription but promotes transcription when the cells are treated with IFN-γ. Furthermore, miR-520G together with mutant-p53 promotes *MYO18B* transcription. Conversely, bFGF suppresses *MYO18B* mRNA irrespective of CEBPB, miR-520G overexpression or IFN-γ treatment. Finally high *MYO18B* expression reflects poor prognosis while high *MYL5* or *MYO1B* expression reflects better survival of HCC patients. Thus, we identified a network of positive and negative regulators of *MYO18B* mRNA expression which reflects the survival of HCC patients.

## Introduction

Hepatocellular carcinoma (HCC) is one of the most lethal cancer types and accounts for ~ 42,220 new cases, and ~ 30,200 deaths in United States alone in 2018 (for Liver & intrahepatic bile duct)^1^. Cirrhosis of the liver is a major risk factor for HCC^2,3^ and obesity is thought to play a role in this context^4^. CCAAT/enhancer binding protein-β (CEBPB) is a major regulator of obesity and also regulates inflammation in the context of obesity^5–10^. Acute myopathy is a common characteristic feature of cirrhotic liver^11^. Mutations or loss of expression of *MYO18B* (Myosin-18B gene, located in chromosome-22) is linked to myopathy^12,13^. *MYO18B* has also been shown to promote progression of HCCs^14^. Therefore, a potential link between obesity, inflammation, myopathy and cirrhosis is evident but not understood in the context of myosin-18B. Myosin-18B could also be targeted by transcriptional regulation and the signaling pathways and components involved in the regulation of *MYO18B* at mRNA level are not yet known. Understanding the signaling pathways and components involved in positive and negative regulation of *MYO18B* transcription is therefore necessary to understand the basics of myosin-18B related progression of HCCs.

A report indicates that *MYO18B* gene is expressed along with chromosome-19 micro-RNA cluster (C19MC) and cancer testis antigens in HCCs^15^. C19MC is a cluster of 46 miRNAs located at chr19q13.42^16,17^. C19MC miRNAs have been implicated in multiple cancer types such as breast cancer^17^, embryonal tumors with multilayered rosettes (ETMRs)^18^, infantile hemangioma^19^, thyroid adenomas^20^, testicular germ cell tumors^21^, parathyroid tumors^22^ , undifferentiated embryonal sarcoma of the liver (UESL)^23^, including hepatocellular carcinoma^24–26^. However, the role of C19MC miRNAs in the context of *MYO18B* gene transcription is not known to date.

Using human hepatocellular carcinoma (HCC) patient data here we show that, C19MC overexpression is tightly linked to *MYO18B* mRNA expression in patients who harbor transcription incompetent p53. In p53 defective Hep3B cells, the expression of *MYO18B* is suppressed by interferon-γ (IFN-γ) and that the presence of C19MC miRNA-520G reverses this suppressive effect to promote the expression of *MYO18B* mRNA. Stable overexpression of *CEBPB* mimics the effect of miR-520G in promoting *MYO18B* mRNA expression. Furthermore, wild-type and mutant p53s promote the expression of *MYO18B* mRNA in the presence of miR-520G. On the other hand, basic Fibroblast Growth Factor (bFGF) suppresses MYO18B mRNA expression irrespective of IFN-γ treatment, *CEBPB* overexpression or miR-520G expression. Thus our study significantly expose the transcriptional regulatory network of *MYO18B*, which in future will help to study the role of these signaling pathways in myopathy, cirrhosis of the liver, and the development of hepatocellular carcinoma.

## Results

### High MYO18B mRNA expression is correlated with C19MC overexpression and poor survival in hepatocellular carcinoma

**Figure 1:**
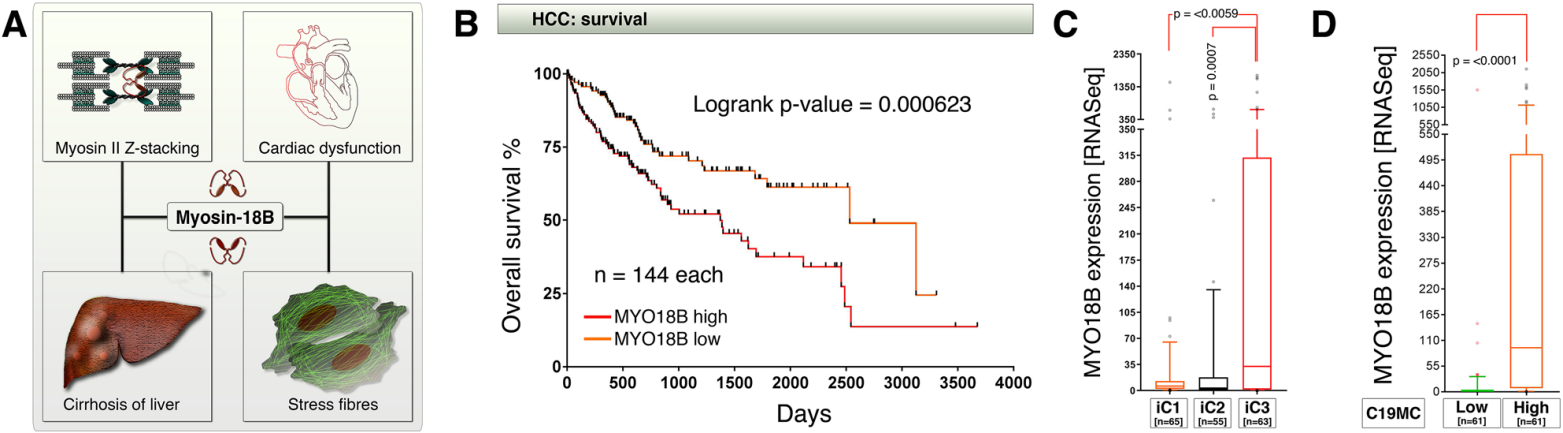
*MYO18B* mRNA expression in integrated cluster iC3 and C19MC miRNA expressing tumors reflects poor prognosis in hepatocellular carcinoma. (**A**) Schematic showing the known involvement of MYO18B in various pathophysiological conditions. (**B**) TCGA-HCC survival data showing the association of high *MYO18B* mRNA expression to poor prognosis of patients. The upper and lower percentages used to extract survival data from Oncolnc database was 40%. (**C**) TCGA RNA-seq data showing the higher expression of *MYO18B* mRNA in iCluster-3 (iC3) compared to iClusters-1 and 2. The data is represented as 10–90% box and whisker plot with whiskers of 75% transparency. (**D**) Integrated iCluster RNA-seq dataset to miRNA-seq dataset showing the increased expression of MYO18B in C19MC miRNA expressing HCCs. Note: The C19MC high versus low grouping was based on cumulative expression of all 46 C19MC miRNAs. The data is represented as 10–90% box and whisker plot with whiskers of 75% transparency.

Deregulated expression of Myosin-18B is linked to HCC progression, stress fiber formation, cirrhosis of liver, and cardiac dysfunction, through defects in myosin-II z-stack formation of muscle fibers (Fig. 1A). In hepatocellular carcinoma patients, high *MYO18B* mRNA expression is significantly associated with poor survival (Fig. 1B). We next examined whether *MYO18B* mRNA expression is associated with any of the integrated molecular classification clusters (iClusters) using TCGA iCluster dataset^27^. *MYO18B* mRNA was significantly enriched in iCluster-3 (Fig. 1C), a cluster known to harbor most p53 defects in HCCs^27^. Furthermore, examination of integrated RNA-seq and miRNA-seq data revealed that, *MYO18B* mRNA is significantly expressed in tumors with high C19MC miRNA expression (Fig. 1D). Taken together, these data demonstrate that, high *MYO18B* mRNA expression is correlated with C19MC overexpression and poor survival in iCluster-3 of HCCs.

### Genomic structure of *MYO18B* gene enhancer reveals multiple CEBPB binding sites

**Figure 2:**
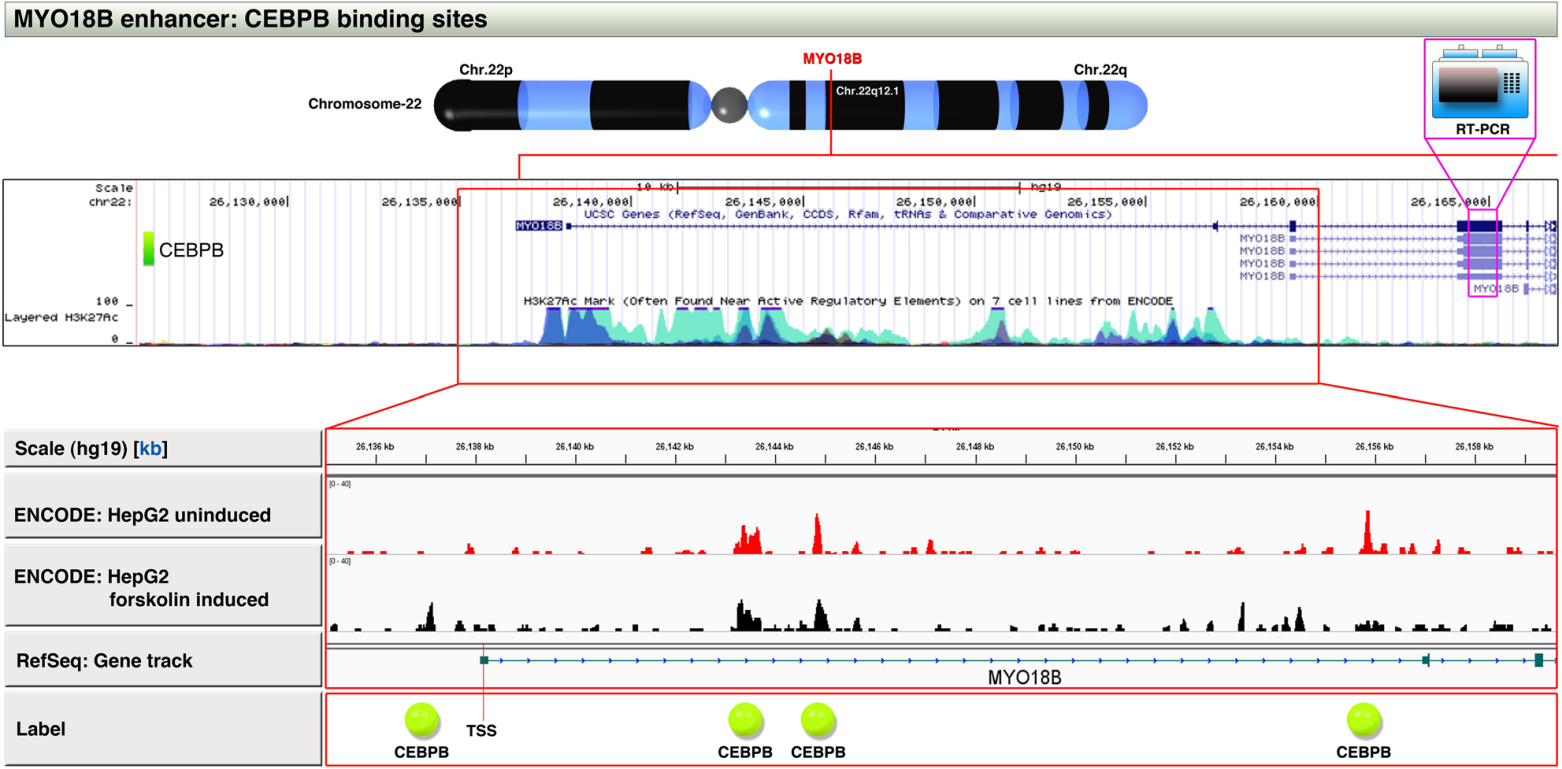
*MYO18B* enhancer region CEBPB binding sites and selection of exons for RT-PCR analysis. The *MYO18B* gene location is shown in the chromosome locus “22q12.1” by a red vertical line. The enhancer region of *MYO18B* gene with H3K27Ac mark (Chr22: 26,135,000–26,160,000 [hg19]) in UCSC Genome Browser track (blue peaks) was focused to show CEBPB binding at this region using ENCODE CEBPB ChIP-seq HepG2 data (red and black peaks). Note the three CEBPB binding sites (indicated by red peaks) within the enhancer region (indicated by red box) in uninduced HepG2 cells and a notable fourth binding site close to transcription start site (TSS) in forskolin induced HepG2 cells. One another CEBPB site was far upstream to enhancer region (indicated by green shaded box). The exon chosen for RT-PCR analysis is indicated at exon-3 of long isoform.

To understand the transcriptional cause for the high expression of *MYO18B* mRNA in iCluster-3, we examined the 5′-regulatory region of *MYO18B* gene in UCSC genome browser. *MYO18B* gene is located on Chr22q12.1 and has a very strong enhancer marked by H3K27Ac (chr22:26,137,306–26,162,170: hg19) (Fig. 2). Transcription factor ChIP-Seq data from UCSC genome browser (ENCODE) revealed numerous transcription factor binding sites within this enhancer which includes CEBPB, p53, Myc, Max, GATA-2 and others (data not shown), but we focused our attention on CEBPB because of the following reasons: (i) CEBPB has the capability to regulate enhancers in liver environment^28^ (ii) CEBPB is tightly linked to obesity^5^ and (iii) CEBPB sites were also present at C19MC region on chromosome-19^17^. *MYO18B* has 3 CEBPB binding sites within its enhancer region and a fourth CEBPB binding site located upstream to the enhancer region (Fig. 2). CEBPB is capable of binding to these regions as evaluated by examining ENCODE ChIP-seq data (Fig. 2). Notably, CEBPB binds to a fourth site close to transcriptional start site (TSS) upon forskolin induction (Fig. 2). While the CEBPB sites may regulate different isoforms of MYO18B mRNAs, we chose exon-3 of the longest isoform for expression analysis by RT-PCR because it is shared by multiple isoforms of MYO18B mRNAs (Fig. 2). Taken together these data reveal that, the *MYO18B* gene harbors a strong enhancer with four CEBPB binding sites.

### Hsa-miR-520G-3p remodels IFN-γ but not bFGF signaling to regulate *MYO18B* transcription

**Figure 3:**
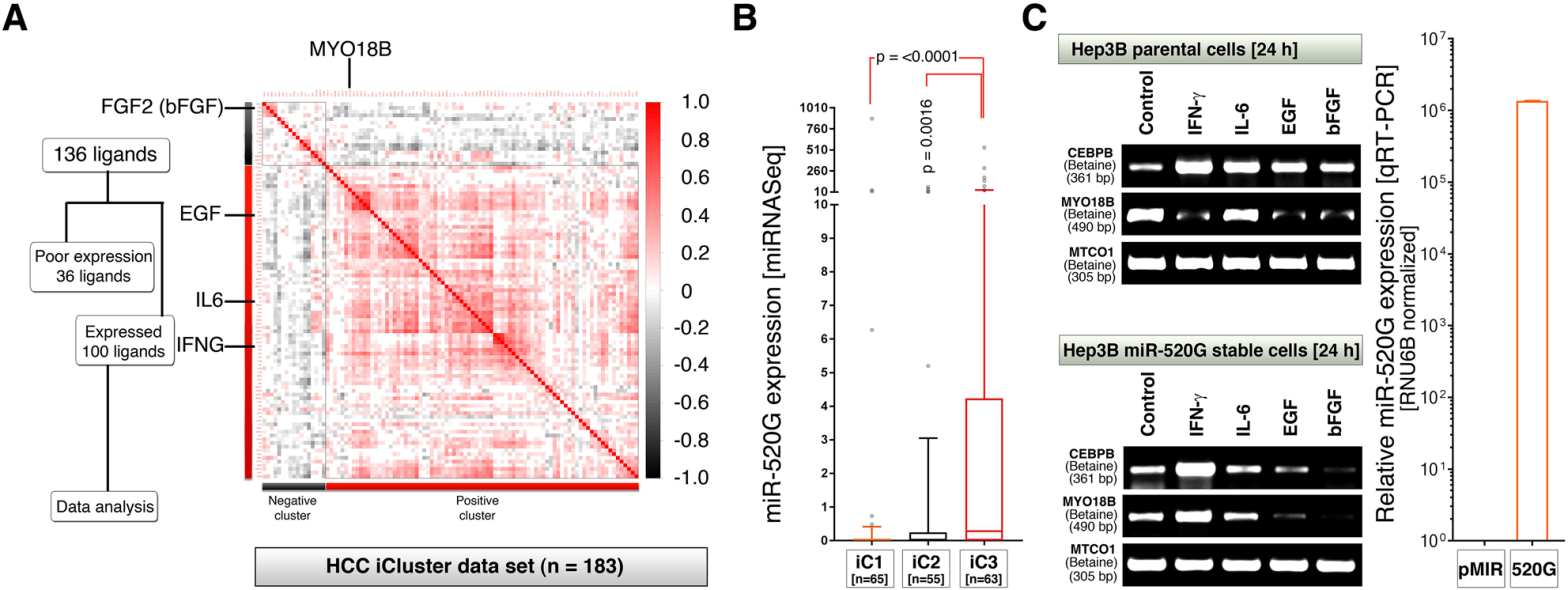
Interferon- γ co-operates with miR-520G to promote *MYO18B* transcription but bFGF antagonizes it. (**A**) One hundred ligands (mRNAs of cytokines and chemokines) that are expressed at mRNA level in hepatocellular carcinomas in complete iCluster dataset (as some samples in iCluster-1 and 2 also expressed good quantities of MYO18B: see Fig. 1C) were subjected to correlation analysis to *MYO18B* RNA. Note that the cytokines such as IFNG, IL6, and EGF forms positive correlation to *MYO18B* whereas bFGF is negatively correlated. (**B**) 10–90% box and whisker plot with whiskers of 75% transparency, showing the expression of C19MC miR-520G is significantly elevated in iCluster-3 (iC3). (**C**) Hep3B cells treated with 1 nM of each cytokines for 24 h and the expression of *CEBPB* and *MYO18B* mRNAs were examined by RT-PCR (gel images). Note that *MYO18B* expression is suppressed by IFN- γ, EGF and bFGF in parental Hep3B cells (top panel) whereas *MYO18B* is promoted by IFN- γ in miR-520G stably overexpressed Hep3B cells (bottom panel). Real-time quantitative PCR (right bar graph) showing the level of miR-520G-3p expression in control (pMIR) and miR-520G stably transfected cells.

Although *MYO18B* has multiple transcription factor binding sites in addition to CEBPB and a strong enhancer, additional signals are likely needed to activate transcription from *MYO18B* gene. Therefore we screened the mRNA expression of cytokines and chemokines for correlation to *MYO18B* mRNA expression in HCCs. We chose the cytokines bFGF, IFN-γ, EGF and IL-6 that are related to myopathy and or cirrhosis^29–32^, and found that, bFGF is negatively correlated and the remaining three cytokines are positively correlated to *MYO18B* mRNA expression (Fig. 3A). To understand the effect of C19MC miRNA expression on *MYO18B* mRNA expression we chose miR-520G which is known to promote drug resistance in cancer cells^33,34^. Analysis of miR-520G in HCCs revealed that this miRNA is expressed more in iCluster-3 (Fig. 3B), a cluster also expresses more MYO18B mRNA (Fig. 1C).

Treatment of Hep3B cells with 1 nM each of IFN-γ, IL-6, EGF and bFGF promoted *CEBPB* mRNA expression while IFN-γ, EGF and bFGF suppressed *MYO18B* mRNA expression (Fig. 3C). However, in miR-520G stably transfected cells, IFN-γ promoted both *CEBPB* and *MYO18B* mRNAs whereas EGF and bFGF treatment downregulated both *CEBPB* and *MYO18B* mRNAs (Fig. 3C). The effect of *CEBPB* promotion was stronger in IFN- γ treated conditions compared to the other cytokines tested (Fig. 3C). The effect of *MYO18B* mRNA suppression was stronger in bFGF treated conditions (Fig. 3C), which stand in line with the negative correlation of *FGF2* with *MYO18B* in HCC patients (Fig. 3A). Although IL-6 could promote CEBPB in Hep3B untransfected cells, it could not promote CEBPB mRNA in miR-520G stable cells (Fig. 3C). Taken together these results demonstrated that, miR-520G remodels IFN- γ signaling to promote *MYO18B* transcription and that bFGF negatively regulate *MYO18B* mRNA expression.

### CEBPB mimics the effect of miR-520G in *MYO18B* mRNA expression but bFGF counteracts it

**Figure 4:**
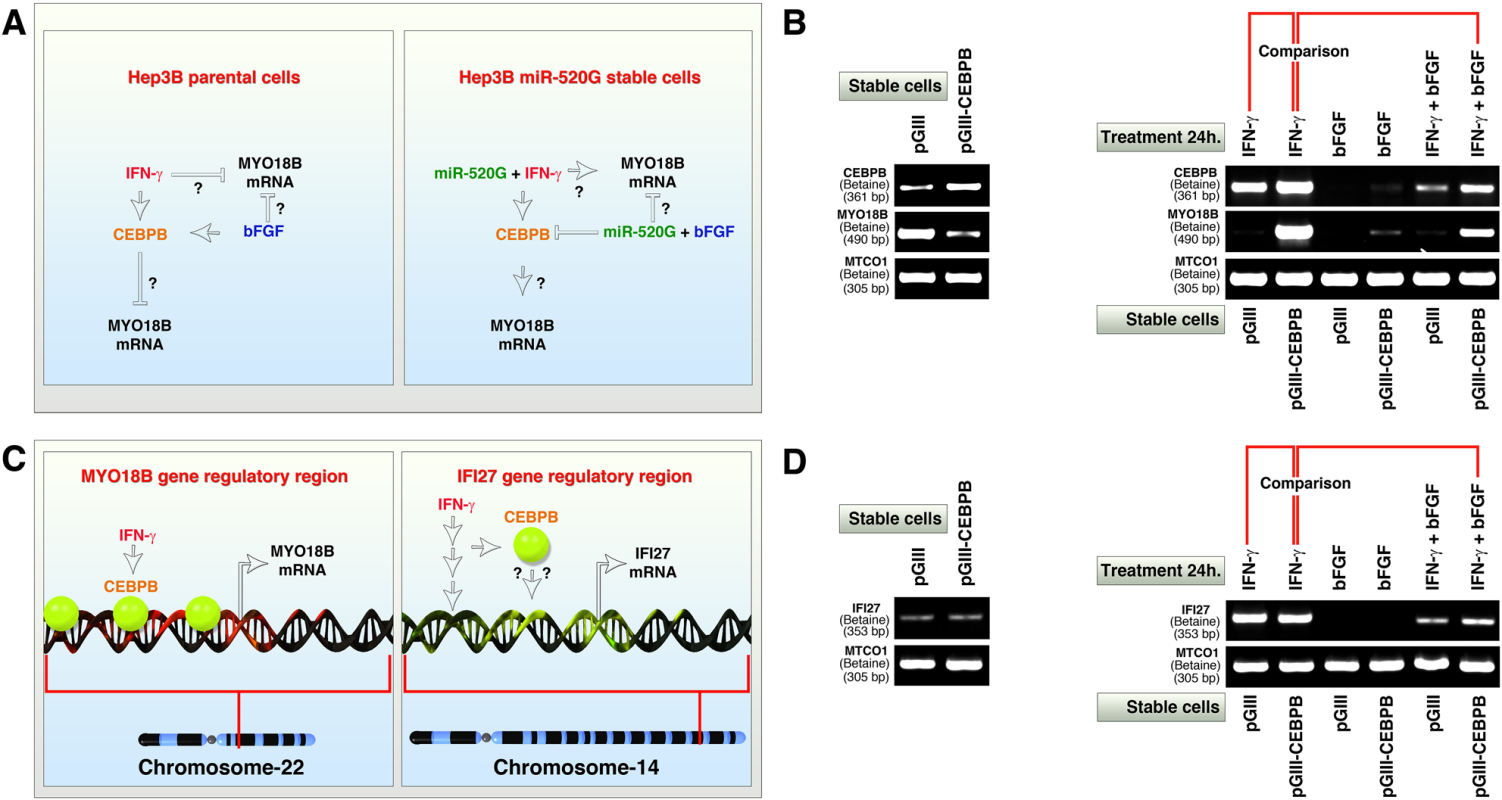
CEBPB-LAP recapitulates the promotion of *MYO18B* transcription by IFN- γ but fails to do so in *IFI27* gene which lacks CEBPB binding site. (**A**) Schematic showing the potential role of CEBPB that might play a pivotal role in determining the differences in *MYO18B* transcription in parental Hep3B cells versus miR-520G stably overexpressed Hep3B cells. (**B**) Empty vector (pGIII) and CEBPB-LAP isoform (pGIII-CEBPB) stably transfected Hep3B cells show reduction in *MYO18B* mRNA expression (left panel) but show increased expression when treated with IFN-γ. All cytokine treatments were at 1 nM final concentration for 24 h. The comparison line is to show the reduction in *MYO18B* mRNA between IFN-γ and IFN-γ  + bFGF treated conditions. Note the complete suppression of CEBPB mRNA in bFGF treated conditions (see discussion for more details). (**C**) Schematic showing the experimental plan to test the role of CEBPB in IFN-γ stimulated promotion of the transcription of *MYO18B* that contains CEBPB binding sites versus another IFN-γ target gene, IFI27 that lack CEBPB binding site. Also see Supplementary figure-2 to see lack of considerable CEBPB binding at IFI27 gene regulatory region in HepG2 CEBPB-ChIP-seq data. (**D**) Empty vector (pGIII) and CEBPB-LAP isoform (pGIII-CEBPB) stably transfected Hep3B cells show no difference in *IFI27* mRNA expression at baseline levels (left panel) or when treated with IFN-γ (right panel). Note: The cDNAs used for panel-**B** and panel-**D** are same and hence the IFN- γ induced changes of *MYO18B* (panel-**B**) and lack of changes in *IFI27* (panel-**D**) are comparable between panels.

We noted a striking correlative upregulation or downregulation of CEBPB with MYO18B mRNA levels in response to IFN- γ or bFGF respectively (Fig. 3C) raising the question that, the CEBPB expression level could mimic the effect of these cytokines or miR-520G (Fig. 4A). Of note, miR-520G overexpression does not alter the mRNA expression of MYO18B/IFNG/bFGF/CEBPB/cytokines receptors compared to control pMIR transfected cells (Supplementary figure 1A). Complete lack of IFNG mRNA expression prompted us to examine whether IFNG gene is deleted in Hep3B cells. However, IFNG was not deleted in Hep3B cells as per copy number data (Supplementary figure 1B). To test whether CEBPB expression level could mimic the effect of the cytokines or miR0520G, we stably overexpressed the LAP-isoform of CEBPB (which is known to promote transcription compared to its short isoform: liver-enriched inhibitor protein (LIP)^35^) in Hep3B cells. Overexpression of CEBPB-LAP isoform itself suppressed MYO18B transcription compared to control empty vector transfected cells (Fig. 4B). Importantly, IFN- γ treatment tremendously promoted MYO18B transcription in CEBPB-LAP overexpressed cells compared to empty vector transfected cells (Fig. 4B). However, bFGF treatment suppressed both CEBPB and MYO18B mRNAs even when co-treated with IFN-γ (Fig. 4B). The reduction of CEBPB mRNA in CEBPB-LAP overexpressed condition suggests that, bFGF promotes the degradation of CEBPB mRNA rather than suppressing transcription from CEBPB promoter because the overexpression vector employs a different promoter (CMV).

To understand whether CEBPB binding site is involved in the promotion of the transcription of IFN-γ target genes, we chose *IFI27* gene which is known to get transcribed in response to IFN-γ^36^ but lack CEBPB binding sites or binding within its enhancer region (Fig. 4C and Supplementary figure-2). We examined the same cDNA set that was used for Fig. 4B (CEBPB-LAP overexpressed and its control) and found that, *IFI27* mRNA was not promoted by mere overexpression of CEBPB or when the CEBPB-LAP overexpressed cells were treated with IFN-γ (Fig. 4D). However, bFGF abolished the expression of IFI27 mRNA or impeded the IFN-γ induced IFI27 mRNA expression. These data demonstrate that, CEBPB binding is required for IFN-γ to promote transcription as IFN- γ could not promote IFI27 mRNA in CEBPB-LAP overexpressed cells.

The data from Figs. 3 and 4 together demonstrates that CEBPB is sufficient to mimic the effect of miR-520G in IFN-γ induced alterations of MYO18B transcription but bFGF suppresses MYO18B mRNA levels irrespective of IFN-γ or CEBPB overexpression.

### Transcription defective p53, increased miR-520G and *MYO18B* expression reflect a lethal phenotype with cellular transformation in HCCs

**Figure 5:**
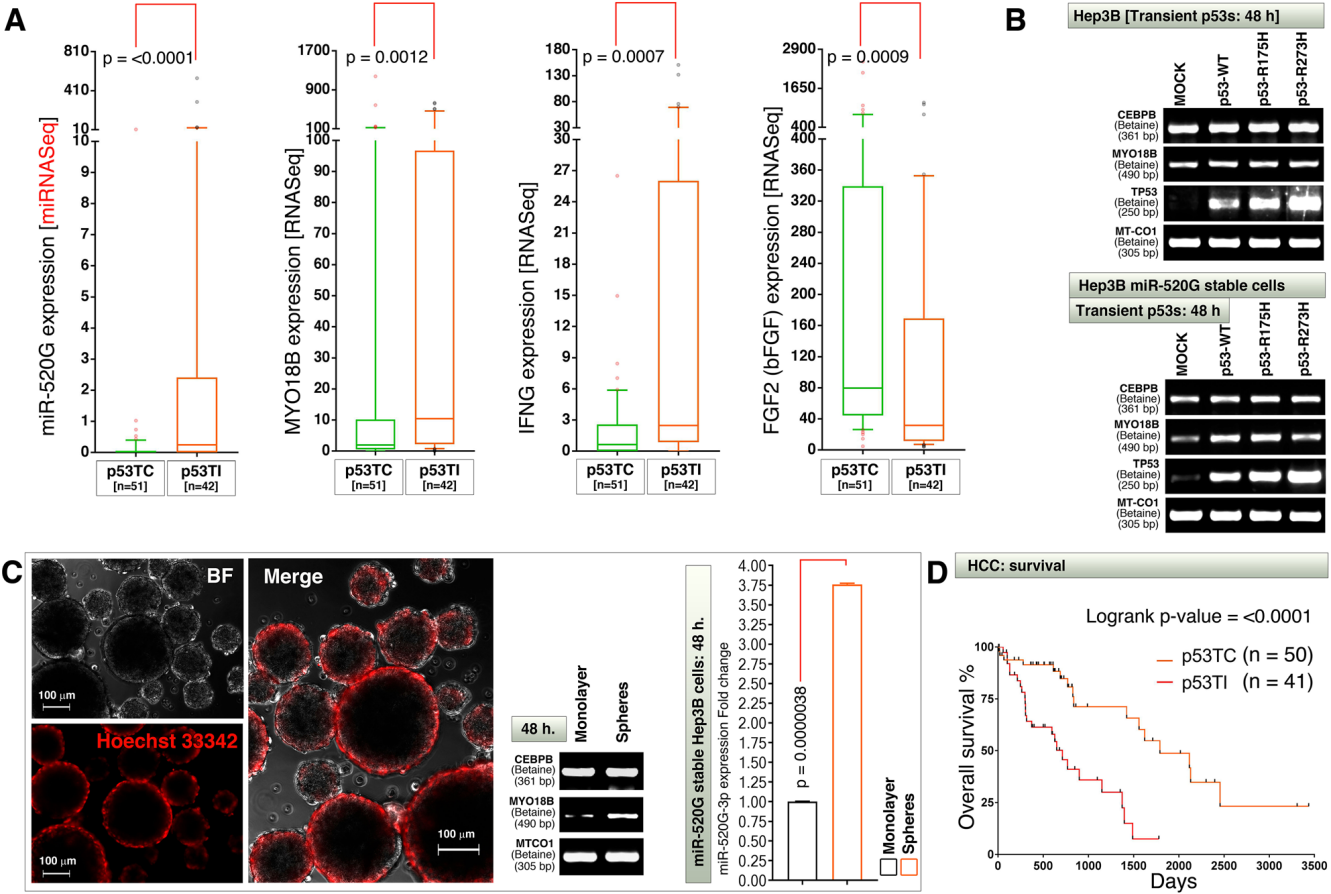
Transcription incompetent p53, increased C19MC miR-520G, IFNG, MYO18B and reduced FGF2 transcription reflects a lethal phenotype in HCCs. (**A**) HCC integrated patient data classified based on p53 transcriptional competence (p53TC) and p53 transcriptional incompetence (p53TI) were examined for miR-520G, *MYO18B*, *IFNG* and *FGF2* mRNA expression. The data are represented as 10–90% box and whisker plots with whiskers of 75% transparency. (**B**) Hep3B parental (top panel) and miR-520G (bottom panel) stably overexpressed cells were transiently transfected with wild-type or mutant p53 plasmids and examined for changes in *CEBPB* or *MYO18B* mRNA expression levels. (**C**) Hep3B cells were grown as monolayers (not shown) or spheres (photo micrographs) for 48 h and examined for changes in *MYO18B* and *CEBPB* mRNA levels (photo micrographs and RT-PCR agarose gel panels). Hep3B stable cells with miR-520G overexpression were cultured as monolayers or spheres for 48 h and examined for miR-520G-3p expression of real-time PCR (bar graph). Note: The high expression of miR-520G in monolayer miR-520G stable cells is set to the value of 1 for fold change calculation purpose. (**D**) Overall survival analysis of HCC patients who display clear p53TC versus p53TI transcription profiles. Survival data was extracted from Oncolnc database and matched to p53TC and p53TI dataset IDs to generate overall survival curves.

We next examined the possible reasons why patients with high MYO18B exhibited poor overall survival. In general, p53 defective tumors are the indication for poor survival and p53 defects can be of one or many of the different types (such as copy number loss, transcriptional repression, degradation at protein level, or gain-of-function due to mutations). Therefore we classified HCCs into p53-transcription competent (p53TC) or p53-transcription incompetent (p53TI) groups using a p53-target gene transcription signature that consists of 30 genes^27^ and integrated this dataset to miRNA-seq data. Interestingly, miR-520G, MYO18B and IFNG RNAs were significantly expressed more in p53-transcription incompetent tumors than in p53-transcription competent tumors (Fig. 5A). On the other hand, bFGF (*FGF2*) mRNA was significantly downregulated in p53-transcription incompetent tumors than in p53-transcription competent tumors (Fig. 5A).

Considering the fact that Hep3B cells harbor p53 defects^37,38^, *MYO18B* is constitutively expressed at mRNA level in this cell line (Fig. 5B). Transient overexpression of wild-type (WT) or gain-of-function mutant p53s (R175H and R273H) did not promote *MYO18B* transcription in Hep3B cells but promoted *MYO18B* transcription in miR-520G stably overexpressed cells (Fig. 5B). Furthermore, sphere formation represents cancer cells with aggressive and transformed phenotype^39–41^, therefore, we examined whether *MYO18B* transcription is altered in monolayer versus sphere forming Hep3B cells. *MYO18B* mRNA is expressed more in sphere forming cells than monolayer cells (Fig. 5C). We further tested the miR-520G expression in miR-520G stably transfected Hep3B monolayer cells versus spheres at 48 h and found that, the spheres accumulate 3.76 (SEM =  ± 0.014) fold higher amount of miR-520G-3p compared to monolayer (Fig. 5C). Survival analysis of p53TC and p53TI tumors revealed that the p53TI patients had significantly poor prognosis than the p53TC patients (Fig. 5D).

Taken together these data demonstrate that transcription defective p53, increased C19MC miRNA-520G expression in patients and transformed state of cells and increased *MYO18B* transcription reflects a lethal phenotype with cellular transformation in HCCs.

### *MYO18B* is negatively correlated to *MYL5* and *MYO1B* expression to reflect survival outcome

**Figure 6:**
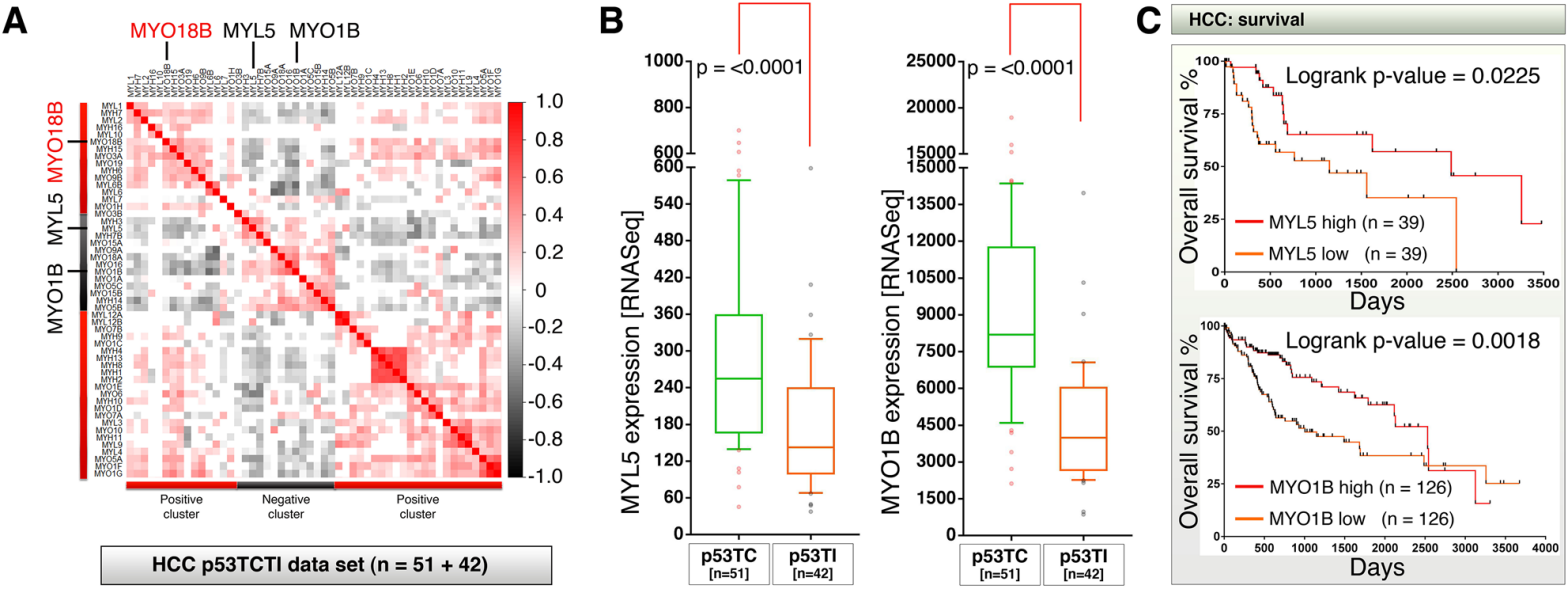
*MYL5* and *MYO1B* mRNAs are negatively correlated to *MYO18B* mRNA expression to reflect better survival outcome in HCC patients. (**A**) A panel of 52 expressed myosin genes were subjected to correlation analysis using p53TCTI RNA-seq dataset of HCC patients. Myosins that did not expressed in any of the p53TCTI dataset were omitted from analysis. Note: *MYL5* and *MYO1B* were negatively correlated to *MYO18B* mRNAs. Insignificant correlations were shown as blank. (**B**) HCC patient RNA-seq data classified based on p53 transcriptional competence (p53TC) and p53 transcriptional incompetence (p53TI) were examined for *MYL5* and *MYO1B* mRNA expression. The data are represented as 10–90% box and whisker plots with whiskers of 75% transparency. (**C**) Overall survival analysis of HCC patients who display clear high and low expression of *MYL5* (top) mRNA or *MYO1B* (bottom) mRNA expression profiles. The upper and lower percentages used to extract survival data from Oncolnc database for MYL5 and MYO1B were 11% and 35% respectively.

*MYO18B* is part of a large family of myosin genes which constitutes both myosin heavy chains and light chains to provide structural organization of cells and tissues such as liver. Cirrhotic liver often show abrupt texture of liver and therefore more myosins may have redundant roles along with or against *MYO18B*. Therefore we next examined whether other family members of myosin-18B positively or negatively correlate with *MYO18B* expression. For this purpose we subjected the 52 myosin family member genes from the p53TC/p53TI RNA-seq expression dataset to correlation analysis and found that, many myosins were positively or negatively correlated to *MYO18B* (Fig. 6A). We focused on two myosins, *MYO1B* and *MYL5* that were negatively correlated to *MYO18B* expression in HCCs and were expressed significantly lower quantities in p53TI tumors compared to p53TC tumors (Fig. 6A,B). This result suggested that, higher expression of these myosins may reflect better survival and p53-transcriptional competence. In line with this, overall survival analysis based on *MYL5* or *MYO1B* revealed that, higher expression of *MYL5* or *MYO1B* is significantly associated with better survival in HCCs (Fig. 6C), which is in contrast to high *MYO18B* expression (Fig. 1B).

Taken all the results together, IFN- γ, CEBPB and C19MC miRNA-520G-mediated high *MYO18B* expression reflects p53 transcriptional defects and poor survival in HCCs whereas, high *FGF2* (bFGF), *MYL5* and *MYO1B* expression reflects p53 transcriptional competence and better survival.

## Discussion

**Figure 7:**
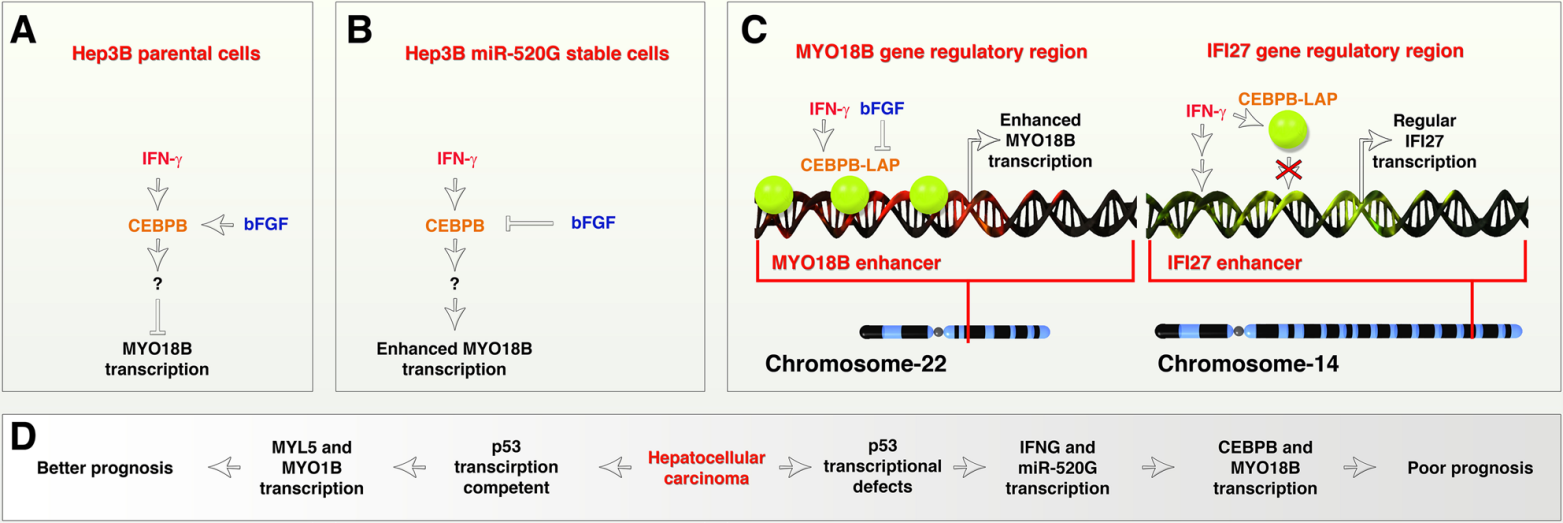
Regulatory network of MYO18B mRNA that reflect the survival outcome in hepatocellular carcinoma. (**A,B**) Schematic showing MYO18B mRNA expression is inhibited by IFN-γ and bFGF through CEBPB (panel-**A**) but promotes the same in C19MC miR-520G stable cells (panel-**B**). (**C**) Schematic showing the requirement of CEBPB binding site within the regulatory regions of target genes to get enhanced transcription by IFN-γ. Note: IFI27 gene regulatory region do not harbor CEBPB binding sites but MYO18B has CEBPB binding sites. (**D**) Overall signaling pathway that reflects survival outcome in HCCs: based on experiments and clinical data.

Cirrhosis of the liver is a major and classical risk factor for HCC^2,3^ and obesity is thought to play a role in this context^4^. Therefore, the pathways that modulate cirrhosis and obesity may play a role in the prognosis of HCC patients. *MYO18B* has been shown to promote progression of HCCs through PI3K/Akt/mTOR pathway^14^. However, the regulation of *MYO18B* is not characterized in detail. Hereby, we show for the first time that, a complex network of IFN-γ, CEBPB (a transcription factor drives obesity through adipogenesis^5,7,8^), miR-520G, and p53-defects co-operatively regulate the expression of *MYO18B* mRNA which in turn reflects the poor survival of HCC patients (Fig. 7). On the other hand we show the interesting negative regulatory aspect of bFGF in counteracting *MYO18B* mRNA expression induced by IFN-γ/CEBPB network (Fig. 7). Presence of CEBPB binding sites is a crucial aspect in the promotion of *MYO18B* mRNA because another IFN-γ target gene *IFI27* failed to get promoted and lacks CEBPB binding site (Fig. 4C,D and Supplementary figure-2). In fact, bFGF may promote the degradation of *CEBPB* mRNA to achieve the negative regulation of IFN-γ-induced *MYO18B* mRNA expression because, bFGF almost silenced CEBPB mRNA expression despite the fact that CEBPB was overexpressed using a CMV promoter (therefore it is not due to repression of original genomic CEBPB promoter alone) (Fig. 4B).

Defects in p53 can result in aggressive phenotype involving cancer stem cell expansion through blebbishield emergency program-mediated cellular transformation/sphere formation^33,39,40,42–52^. Therefore, *MYO18B* expression may not be the direct cause of poor survival in HCCs but it reflects the poor survival due to its association with p53 transcriptional incompetence and associated aggressive therapy resistance and stem-cell expansion phenotypes. The increased expression of *MYO18B* mRNA and miR-520G in spheres compared to monolayer cells supports this notion. High expression of *MYL5* and *MYO1B* mRNAs indicates an opposite outcome compared to *MYO18B* expression in survival of HCC patients which may possibly be mediated by bFGF-induced counteraction of IFN-γ signaling and therefore, detailed studies on the signaling pathways regulating *MYL5* and *MYO1B* are warranted.

Myosin-18B may contribute to proliferation of cancer cells as targeting *MYO18B* expression is linked to skeletal muscle cell proliferation in rheumatoid arthritis^53^. In ovarian and colorectal cancers Myosin-18B is considered as a tumor suppressor^54,55^. However, the C19MC miRNAs and IFN-γ (analogous to inflammatory environment of the cirrhotic liver) in p53 defective background may render it as an oncogene in HCCs as per our data.

In summary, our study identified a complex network of IFN-γ, *CEBPB*, C19MC miR-520G and p53-transcriptional incompetence as positive regulators of *MYO18B* mRNA expression and bFGF as negative regulator of *MYO18B* mRNA expression to reflect the survival outcome of HCC patients.

## Materials and methods

### The cancer genome atlas (TCGA) and iCluster details

LIHC RNA-seq, miRNA-seq data were from TCGA (https://gdac.broadinstitute.org/) and an integrated patient data sub-set was used which is based on the patient IDs of integrated cluster (iC1 + iC2 + iC3 = 183 samples). The integrated iCluster dataset was based on the expression of 528 signature genes (200 + 128 + 200 genes from iC1, iC2 and iC3 respectively) as described previously^27^. The TCGA IDs of iClusters were generously provided by Dr. Lee, Ju-Seog (UT MD Anderson Cancer Center, Houston, TX, USA), Dr. Ronglai Shen (Memorial Sloan Kettering Cancer Center, New York, NY, USA), Dr. David Wheeler (Baylor College of Medicine, Houston, TX, USA) and Dr. Lewis R. Roberts (Mayo Clinic, Rochester, MN, USA). MYO18B expression was then examined based on iClusters. Expression of miR-520G was examined using miRNA-seq and RNA-seq integrated iCluster dataset.

### C19MC-based grouping of HCC patient data

The TCGA miRNA-seq dataset of LIHC (HCC) was processed to get cumulative miRNA expression of all 46 C19MC miRNA genes (*MIR498, MIR512-1, MIR512-2, MIR515-1, MIR515-2, MIR516A1, MIR516A2, MIR516B1, MIR516B2, MIR517A, MIR517-B, MIR517C, MIR518A1, MIR518A2, MIR518B, MIR518C, MIR518D, MIR518E, MIR518F, MIR519A1, MIR519A2, MIR519B, MIR519C, MIR519D, MIR519E, MIR520A, MIR520B, MIR520C, MIR520D, MIR520E, MIR520F, MIR520G, MIR520H, MIR521-1, MIR521-2, MIR522, MIR523, MIR524, MIR525, MIR526A1, MIR526A2, MIR526B, MIR527, MIR1283-1, MIR1283-2, and MIR1323*) and matched to the HCC-iCluster RNA-seq data set to get RNA-seq and miRNA-seq integrated dataset. MYO18B expression was then examined based on C19MC high versus low groups (n = 61 per group).

### Evaluation of CEBPB binding to *MYO18B* and *IFI27* regulatory regions: ChIP-seq data analysis

The CEBPB ChIP-seq data were accessed from Encyclopedia of DNA Elements (ENCODE)^56^. CEBPB ChIP-seq data sets with or without forskolin induction in HepG2 cells [ENCODE: ENCSR000EEX file: ENCFF000XPP (fold change over control hg19) and ENCSR000BQI file: ENCFF321NDM (fold change over control hg19)] were examined for CEBPB binding at *MYO18B* enhancer region (Chr22:26,135,000–26,160,000, hg19) and visualized using Integrative Genomics Viewer (IGV: BROAD institute, version 2.4.10). The data range was kept constant (40) for both uninduced and forskolin induced tracks. For *IFI27*, same data sets were used with same settings but by focusing on *IFI27* regulatory region (Chr14:94,576,511–94,577,956, hg19). The peaks are comparable qualitatively and quantitatively between *MYO18B* and *IFI27* genes within same tracks however, the uninduced and forskolin induced tracks are comparable only qualitatively but not quantitatively.

### Copy number analysis of *IFNG* in Hep3B cells

Copy number of IFNG gene locus in Hep3B was analyzed using Cancer Cell Line Encyclopedia (CCLE) cell line copy number variation data^57^ within cBioportal platform with built-in IGV visualization option^58^. The default color code applies to the copy number alterations.

### Transcription competent (TC) and transcription incompetent (TI) p53 clustering of iCluster dataset

The integrated RNA-seq iCluster Dataset (described above) was used to generate hierarchical clustering heat map using 30 signature genes that represent p53 transcription competence (includes 10 repressed genes: *FOXM1, CCNB1, CDK1, CCNB2, E2F2, E2F3, PLK1, MYBL2, EZH2, EED,* and 20 transcribed genes: *CDKN1A, AEN, C13orf15, ALDH4A1, ACAD11, PANK1, ESR1, GADD45A, FDXR, DDB2, RPS27L, GADD45B, C6orf138, Fas, EDA2R, SPATA18, PHLDA3, TRIM22, MDM2, ZMAT3*)^27^ and two clusters showing clear differences between p53-repressed and p53-expressed genes across clusters were designated as p53 transcription competent (p53TC) and p53 transcription incompetent (p53TI) clusters. The p53TC group includes 51 patients and p53TI group includes 42 patients. This dataset was integrated with TCGA LIHC (HCC) miRNA-seq dataset and the expressions of C19MC miR-520G, MYO18B, IFNG, FGF2, MYL5, and MYO1B were statistically examined for differential expression using GraphPad Prism software v7.04 (La Jolla, CA, USA).

### Survival analyses and statistics

TCGA LIHC (HCC) survival data for MYO18B were obtained through Oncolnc (https://www.oncolnc.org/) using 40% settings for high and low groups, and matched with RNA-seq dataset sorted based on MYO18B expression values and selected high and low groups (n = 144 each). For MYO1B, survival data was obtained similarly using 35% settings, matched with RNA-seq dataset sorted based on MYO1B expression values and selected high and low groups (n = 126 each). For MYL5, survival data was obtained similarly using 35% settings, matched with RNA-seq dataset sorted based on MYL5 expression values and selected high and low groups (n = 39 each). For p53TC versus p53TI survival analysis the survival data were matched to p53TC (n = 50) and p53TI (n = 41) dataset where the patient number is one less for each group due to non-availability of data.

The survival data were plotted using GraphPad Prism v.7.04 (La Jolla, CA, USA) and the log-rank (Mantel-Cox test) p-values were considered for level of significance. The p-values < 0.05 were considered significant and < 0.001 were considered robust significance.

### Ligand screening and myosin correlation plots, scripts, color code and statistical significance

Correlation plot to screen ligands [100 expressed cytokines and chemokines out of 136 in LIHC (HCC) iCluster dataset: the mRNAs of cytokines or chemokines that are not expressed in any of the samples were omitted (poor expression)] that correlate with MYO18B mRNA expression was generated using R package ‘corrplot’ 0.84 (was built under R version 3.4.4 and R-studio version 1.2.5019. Ref^59^) by using the scripts > cor(); > mat <—cor(); > corrplot(mat, order = "hclust", addrect = 2, method = "color"); > col1 <—colorRampPalette(c("black", "white", "red")); > corrplot(mat, order = "hclust", addrect = 2, method = "color", col = col1(100)), where addrect = 2 was optional; red = positive correlation; black = negative correlation. Significance was calculated using the codes > res1 <—cor.mtest(mat, conf.level = 0.95); > corrplot(mat, order = "hclust", method = "color", addrect = 2, col = col1(100), p.mat = res1$p, insig = "blank") where insignificant correlations were coded white in color. Pearson correlation was used which comes as default option in Corrplot package.

For myosin family correlation with MYO18B, 52 expressed myosin genes were subjected to correlation analysis as described above but using p53TCTI dataset (n = 51 for p53TC and 42 for p53TI) and omitting “addrect = 2” option from the code. Myosins that did not expressed in any of the p53TCTI dataset were omitted from analysis.

The iCluster or myosin datasets from RSEM normalized LIHC (HCC) TCGA RNAseq were log transformed to the base of 10 before generating matrix table in R. The insignificant correlations were coded white and thus white indicates either correlation value = 0 or insignificant.

### Cell line, DNA fingerprinting, plasmids and stable/transient transfections

Human Hep3B cells (ATCC # HB-8064) were cultured in MEM containing L-Glutamine and Sodium bi-carbonate (Sigma #M4655), with 10% FBS (Sigma#F0926), vitamins (Gibco Life Technologies #11120052), sodium pyruvate (Gibco Life Technologies #11360070), non-essential amino acids (Gibco Life Technologies #11140050), and penicillin–streptomycin (Gibco Life Technologies #15140122). The cells were subjected to STR fingerprinting as per institutional/lab standards. The cells were then expanded, and frozen. Fresh vials were used after every 6 months or after ~ 25 passages. The cells in culture were tested for mycoplasma periodically using MycoAlert Kit (Lonza).

Glycerol stocks of mammalian expression vectors such as pMIR-CMV, and pMIR-CMV-520G (CR215781) were purchased from Vigene Biosciences (Rockville, MD USA). The control pLenti-GIII-CMV-RFP-2A-Puro (Cat# LV084) and CEBPB pLenti-GIII-CMV-human-CEBPB-RFP-2A-Puro (corresponding to the LAP isoform) (Cat# LV796074) vectors were purchased from Applied Biological Materials Inc., Richmond, BC, Canada. The lentiviral expression cassettes were used as plasmids for transfection rather than as viruses or with accompanying plasmids to package viruses, because C19MC is a cluster that responds to viral infections. Wild-type p53 (#16434) and mutant p53s p53-R175H (#16436) and p53-R273H (#16439) plasmids under CMV promoter were a gift from Bert Vogelstein^60^. An empty CMV promoter containing plasmid was used as empty control. All plasmids were isolated using Qiagen MIDI prep kit (#12143).

Hep3B cells were stably transfected using plasmids (not viruses in the case of lentiviral plasmids) and Lipofectamine 2000 (Life Technologies # 11668019) and selected using 4 μg/ml puromycin (Invitrogen # A1113803) for 2 months while GFP/RFP positive clones were picked, expanded and frozen. For transient transfections, 1 μg plasmid DNA/10 cm dish was used with Lipofectamine for 12–14 h., in complete MEM, the media were washed off, and the cells were then collected at 48 h. duration (from the time of addition of DNA + Lipofectamine complex to cells).

### Reverse transcriptase PCRs

Parental Hep3B cells were maintained in MEM but the stable cells were maintained in puromycin containing MEM. The stable cells were plated for experiments in MEM without puromycin and treated with 1 nM of cytokines as indicated in figures for 24 h. Total RNA was isolated using TRIZOL reagent (ThermoFisher Scientific #15596026, Waltham, MA, USA) as per manufacturer’s instructions. 20 μl complementary DNA synthesis reactions were done using 1,000 ng RNA and High-Capacity cDNA Reverse Transcription Kit (ABI # 4368814, Foster City, CA, USA) with 1.5 M final concentration of betaine (from 5 M stock: Sigma # B0300-1VL, St. Louis, MO, USA). The temperature conditions were, 25 °C for 10 m, 37 °C for 120 m and 85 °C for 5 m. The cDNAs were then diluted with 30 μl of nuclease free water and then 2.5 μl was used per PCR reaction. For PCR reactions 1 M betaine (final conc.) was used along with regular PCR reaction components. The primer sequences and obtained product sizes were included in Supplementary table-1. All PCR reactions were standardized with a denaturing (95 °C) time of 1 min, annealing temperature of 60 °C (30 s) and 1 min of extension time (72 °C), with 34 cycles. The PCR reactions were run on 2% agarose gels with GeneRuler 100 bp DNA Ladder (ThermoFisher Scientific #SM0243). The gels were imaged using LI-COR Odyssey Fc imager (Lincoln, NE, USA).

### Sphere formation and microscopy

Hep3B cells were plated at high density (500,000 cells/ml) in regular tissue culture 10 cm dish (for monolayer) or in low attachment flasks (for spheres) in complete MEM and cultured for 48hrs with a media change at 24hrs. The spheres were stained with Hoechst-33342 and imaged at 48hrs using Zeiss Observer.Z1 microscope equipped with Axiocam 503 mono (Zeiss) camera. The individual channel images of Hoechst-33342 were pseudo-colored to red, merged with bright field and exported using ZEN 2.3 Pro software (Carl Zeiss Microscopy, GmbH, 2011, Blue edition). The final composite was done using Adobe Photoshop CS5 (Adobe Systems Inc., San Jose, CA, USA). Similar experiments were performed to collect monolayer cells and spheres for RNA isolation for RT-PCR/qRTPCR analysis using Hep3B parental cells or miR-520G stably transfected cells.

### Quantitative real-time PCRs

RNAs were isolated from pMIR or pMIR-520G stably transfected monolayer cells or spheres at 48 h using miRNeasy Mini Kit (Qiagen #217004, Germantown, MD, USA). RNAs were quantified using Nanodrop, and 250 ng RNAs were subjected to cDNA synthesis using Multiscribe reverse transcriptase with RNAse inhibitor, 10X buffer, dNTPs, (ABI, Cat # 4366596) and RT TaqMan™ Primers (RNU6B Control Assay: Assay ID: 001093 (Cat # 4427975), hsa-miR-520 g-3p : Assay ID: 001121 (Cat # 4427975). The cDNAs were then subjected to real-time PCR reactions in triplicates using respective primers with probes and Taqman master mix. The data were normalized using RNU6B and comparative Ct (Δ ΔCt) method was used to compute the relative expression of miRNAs after normalizing with RNU6B values. Statistical significance was calculated in Microsoft Excel (2010) using t-test, two-tailed distribution, two-sample unequal variation option. The results and standard error of mean (SEM) were then plotted using GraphPad Prism software (v7.04; La Jolla, CA, USA).

### RNA-seq evaluation of genes of interest

RNAs from stable miR-520G and pMIR control cells were isolated using miRNeasy Mini Kit (Qiagen #217004, Germantown, MD, USA), with an on-column RNAse free DNAse (Qiagen # 79254) digestion as per manufacturer’s protocol. RNA-seq was then performed in quality control tested RNAs using the NuGen Ovation RNA-seq FFPE System (PN 7150-08) to prepare the libraries and were run on the Illumina NextSeq 500 with a 76-base paired-end read. The adapter reads were trimmed using Cutadapt (v1.8.1) and raw reads were then aligned to human genome (build: hg19) using STAR (v2.5.3a). Gene expression was evaluated as read count at gene level with HTSeq (v0.6.1) and Gencode gene model v28. Gene expression data were then normalized using DEseq2. The genes of interest were then visualized using Microsoft Excel (2010).

### 3D graphics

3D chromosomes, 3D DNA, images were generated using Lightwave Modeler v11.6.3 and rendered using Lightwave Layout v11.6.3 (NewTek Lightwave San Antonio, TX, USA) and composited using Adobe Photoshop CS5. Other graphic images were created using Adobe Photoshop CS5 (Adobe Systems Inc., San Jose, CA, USA).

### Statistical analyses

Frequency distribution 10–90 percentile type box & whisker-plots and statistical analyses were done using GraphPad Prism software (v7.04; La Jolla, CA, USA). In box & whisker -plots the whiskers are aligned and color set to 75% transparency. For patient group versus group statistical significance analysis (box & whisker plots), unpaired, non-parametric Mann–Whitney test was used. For real-time PCRs student’s t-test was performed with two tail, two-sample unequal variance options in Microsoft Excel 2010. Throughout the study the p-value of 0.05 was considered significant and for frequency distribution box & whisker-plots, p-values < 0.001 were considered as robust significance. The ‘n’ for TCGA data analyses were indicated in figures.

## Supplementary information

Supplementary information.
