# Supplementary material for: Regulation of MYO18B mRNA by a network of C19MC miRNA-520G, IFN-γ, CEBPB, p53 and bFGF in hepatocellular carcinoma
Source: Sci Rep. 2020 Jul 23;10:12371. doi: 10.1038/s41598-020-69179-5 (PMC7378193; doi:10.1038/s41598-020-69179-5)
Supplement: Supplementary file 1 — Supplementary information. [file 41598_2020_69179_MOESM1_ESM.pdf]

Regulation of MYO18B mRNA by a network of C19MC miRNA-520G, IFN-γ, CEBPB, p53 and bFGF in hepatocellular carcinoma

Goodwin G. Jinesh, Marco Napoli, Hayley D. Ackerman, Payal M. Raulji, Nicole Montey, Elsa R. Flores, and Andrew S. Brohl

Legends for supplementary figures

Supplementary figure 1. Overexpression of miR-520G does not alter the expression of test subject mRNAs or their receptors

A, Hep3B cells stably overexpressed with miR-520G or its empty vector pMIR were subjected to RNA-seq. Normalized RNA-seq read data from these cells were used to examine the changes in *IFNG*, *FGF2*, or their receptors, *CEBPB* and *MYO18B*. **B,** Note the lack of expression of *IFNG* in panel-A suggestive of potential deletion but was excluded (not deleted) by examining Hep3B copy number variation of IFNG locus at chromosome-12 (hg19) using cBioportal IGV copy number data of Hep3B cells.

Supplementary figure 2. *IFI27* enhancer region has no considerable CEBPB binding sites

The *IFI27* gene location is shown in the chromosome locus "14q32.12" by a red vertical line. The enhancer region of *IFI27* gene with H3K27Ac mark (Chr14:94,576,511-94,577,956 [hg19]) in UCSC Genome Browser track (blue/purple peaks) was focused to examine CEBPB binding at this region using ENCODE CEBPB ChIP-seq HepG2 data (red and black peaks). Note that neither in uninduced HepG2 cells nor in forskolin induced HepG2 cells CEBPB exhibited considerable binding to *IFI27* enhancer/promoter region.

Supplementary figure 3. Original RT-PCR gels of main figures. Part-1

Supplementary figure 4. Original RT-PCR gels of main figures Part-2

Legends for supplementary table

Supplementary Table-1 List of primer sequences and PCR conditions

The primers used for the RT-PCR reactions, were listed in supplementary table-1 with annealing temperatures (Ta) and the usage of betaine for PCR reactions as most of the targets such as CEBPB are highly GC rich in nature.

Supplementary figure - 1

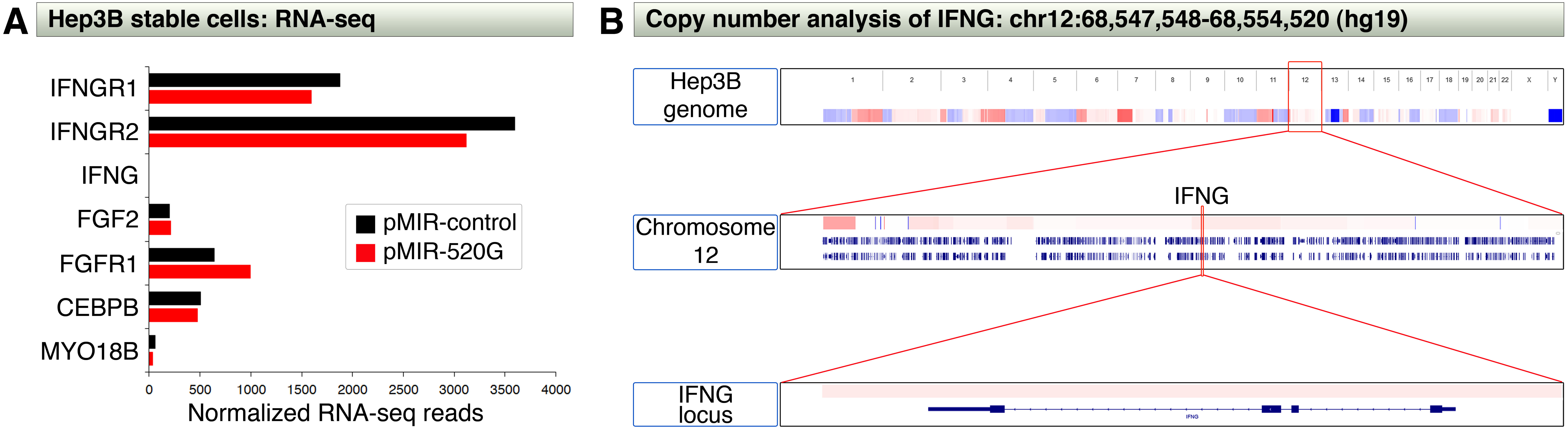

Supplementary figure - 2

IFI27 enhancer: No CEBPB binding

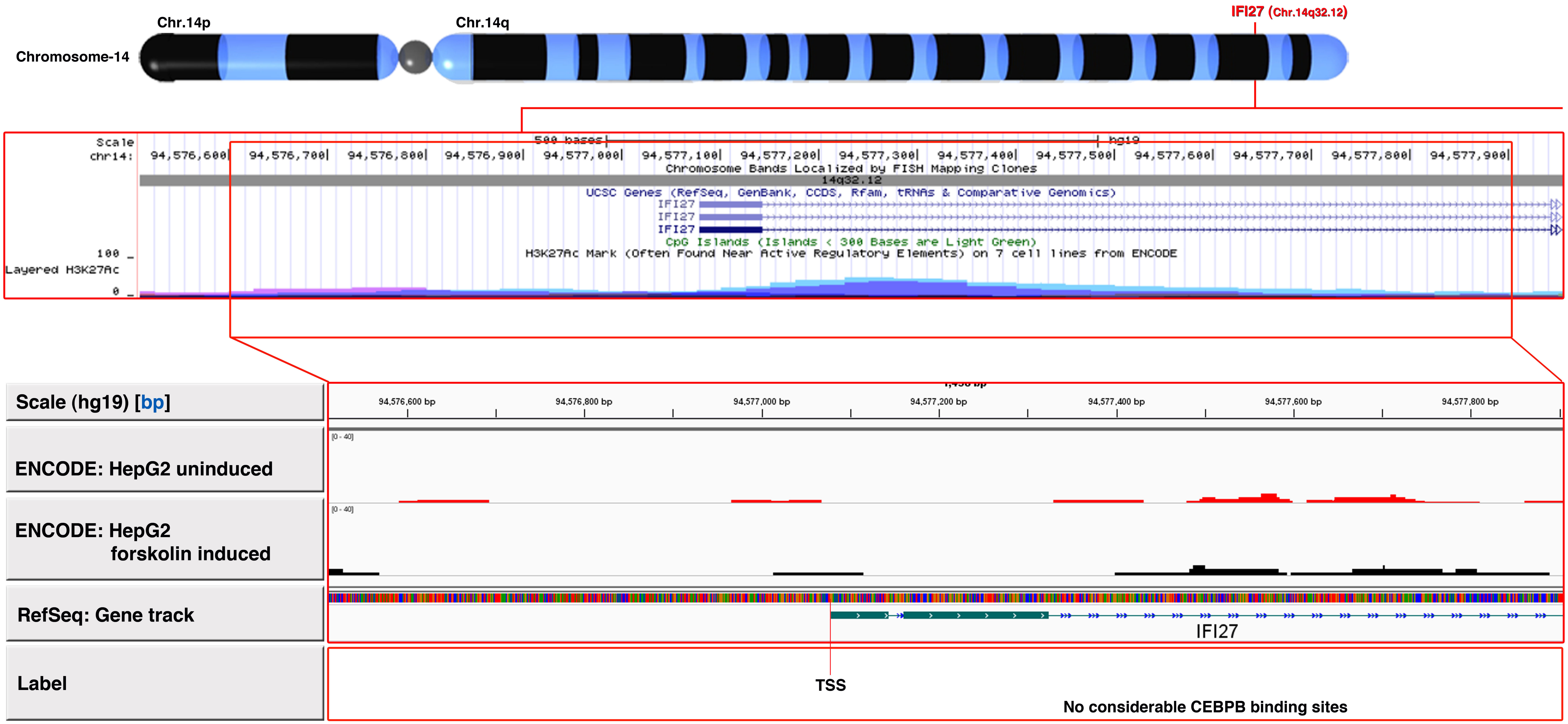

Supplementary figure-3

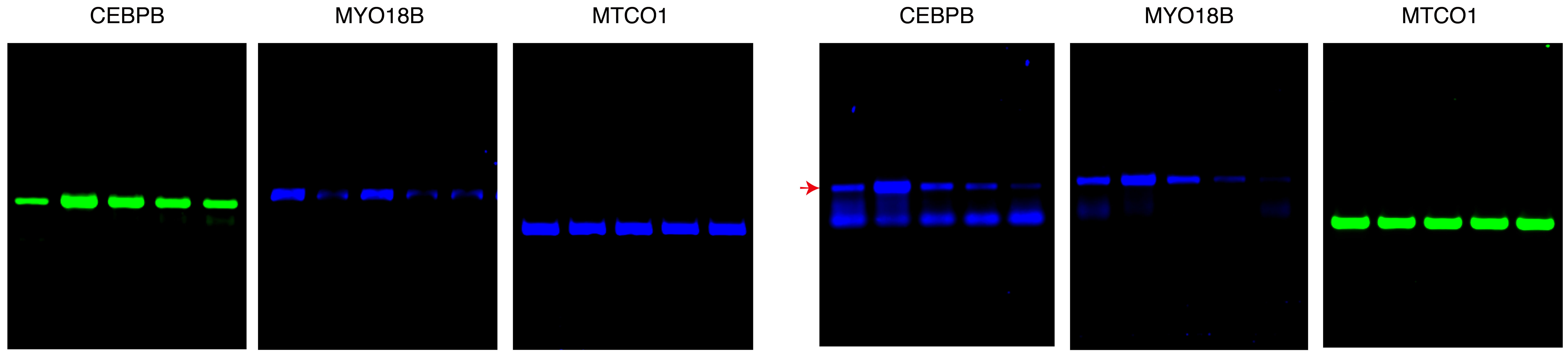

Figure-3c top panel

Figure-3c bottom panel

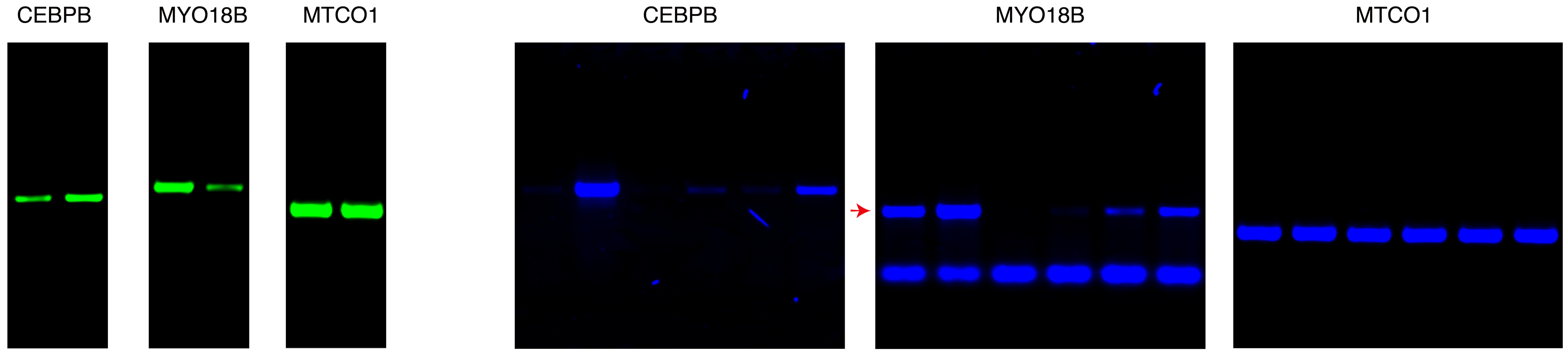

Figure-4b left panel

Figure-4b right panel

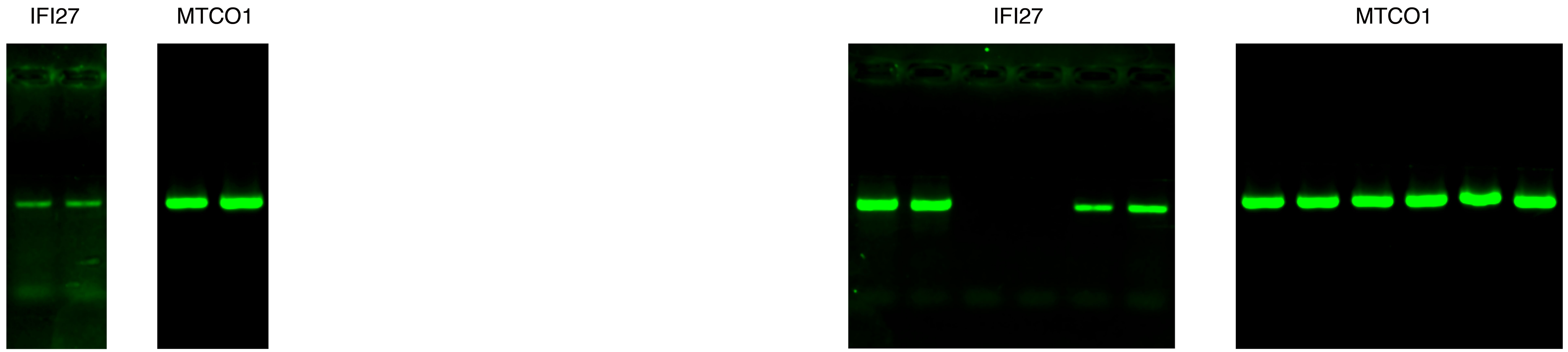

Figure-4d left panel

Figure-4d right panel

Supplementary figure-4

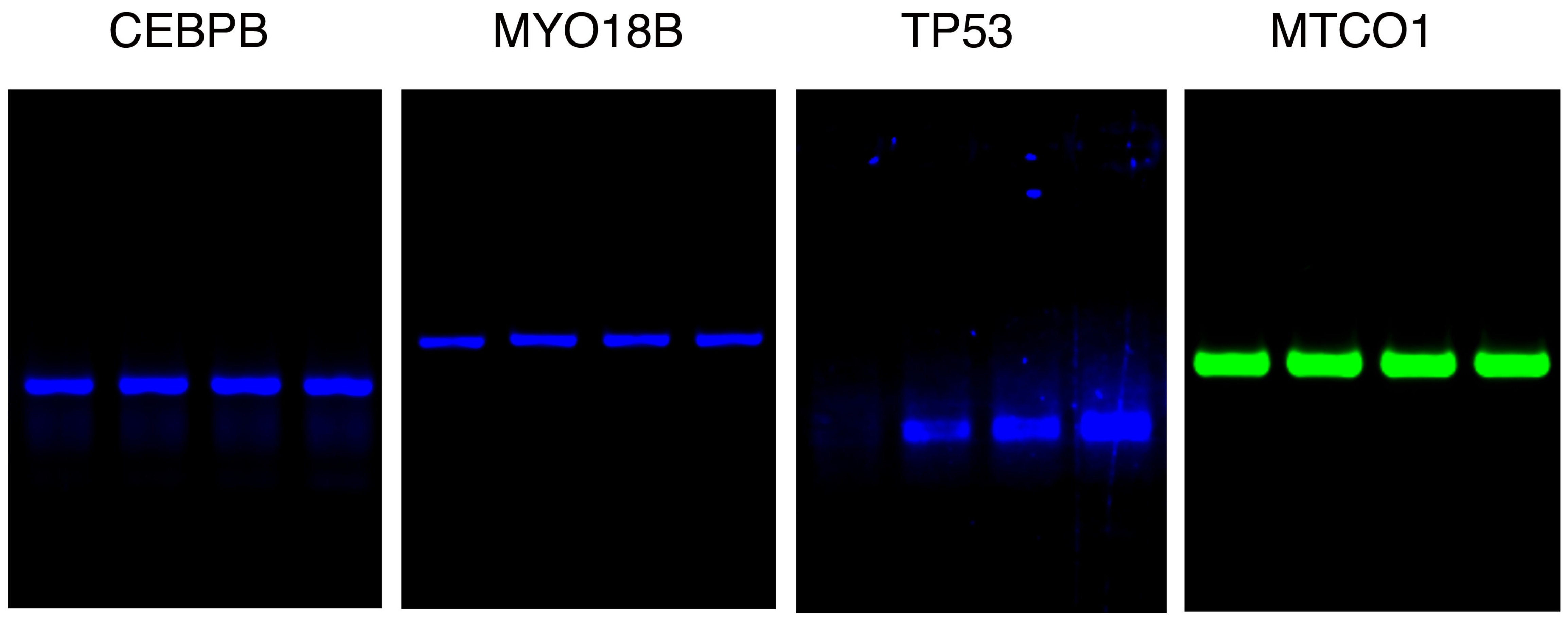

Figure-5b top panel

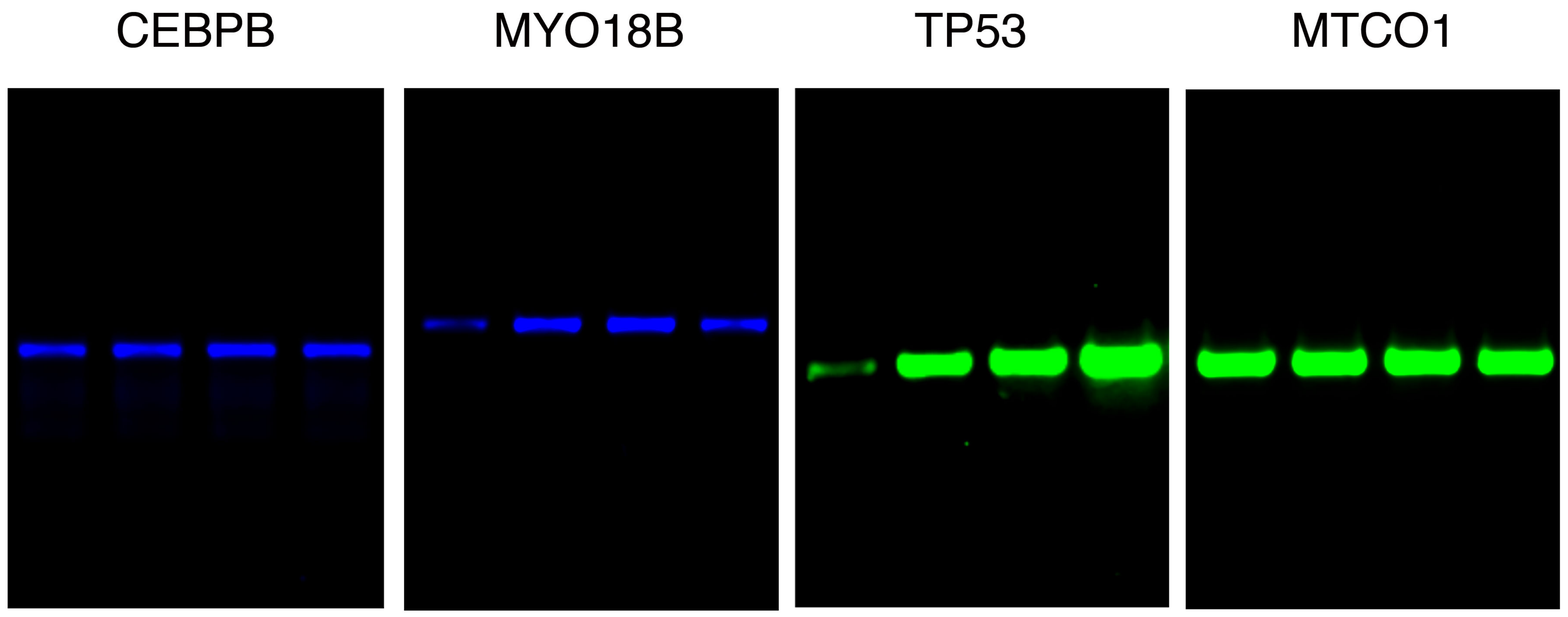

Figure-5b bottom panel

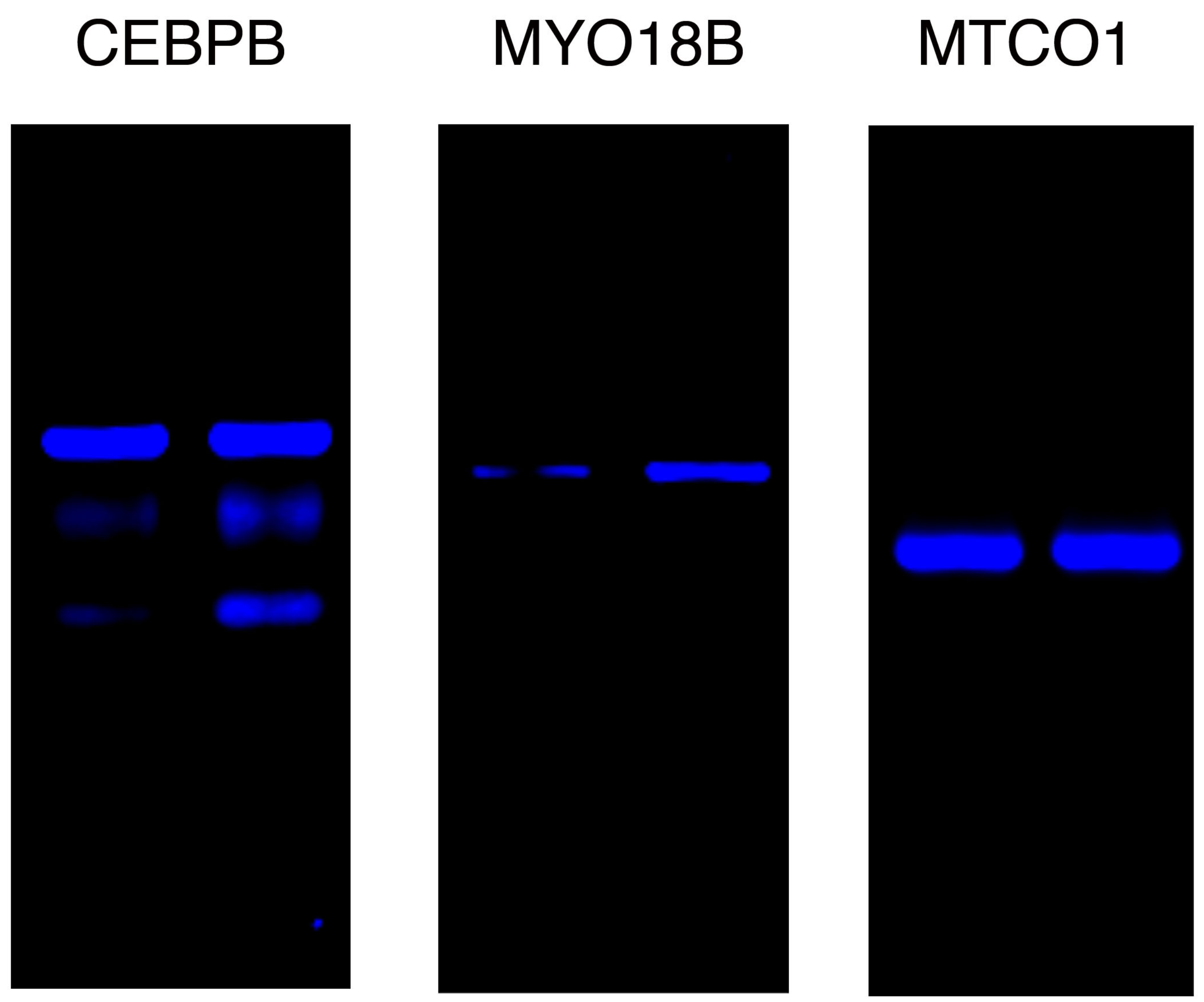

Figure-5c panel

Supp Table-1	**RT-PCR**				
Gene target	Forward primer sequence	Reverse primer sequence	Product size	Ta	Betaine used for PCR [Final conc.]
CEBPB	5'-AACGCCTGGTGGCCTGGGACCC-3'	5'-AAGAGGTCGGAGAGGAAGTCGTGG-3'	361 bp	60°C	1.5M for cDNA and 1M for RT-PCR
MYO18B	5'-ACCCAACAGCAAGTCCAGCAGTGG-3'	5'-ACCCTCCTCGCCTTTTTTCAGTGC-3'	490 bp	60°C	1.5M for cDNA and 1M for RT-PCR
IFI27	5'-TCTGCTCTCACCTCATCAGCAGTG-3'	5'-AACCTCGCAATGACAGCCGCAATGG-3'	353 bp	60°C	1.5M for cDNA and 1M for RT-PCR
TP53-Exon-3	5'-ATGATTTGATGCTGTCCCCGGACG-3'	5'-CGTGCAAGTCACAGACTTGGCTG-3'	250 bp	60°C	1.5M for cDNA and 1M for RT-PCR
MTCO1	5'-ATGAGCTGGAGTCCTAGGCACAGC-3'	5'-AACCTGTTCCTGCTCCGGCCTCC-3'	305 bp	60°C	1.5M for cDNA and 1M for RT-PCR
